# BS Dataset: A Tailor-Made Urban Road Pothole Dataset for Real-Time Detection and Safety-Oriented Monitoring

**DOI:** 10.3390/s26165267

**Published:** 2026-08-20

**Authors:** Roberto Benedetti, Valerio Bortolotto

**Affiliations:** 1 Department of Computer, Control, and Management Engineering, Sapienza University of Rome, 00185 Roma, Italy; 2 Bridgestone Europe NV/SA—Italian Branch Technical Center Europe, Via del Fosso del Salceto 13/15, 00128 Rome, Italy; valerio.bortolotto@bridgestone.eu

**Keywords:** road monitoring, pothole detection, computer vision, deep learning, multimodal dataset

## Abstract

Road surface hazards remain a persistent concern for vehicle safety, passenger comfort, and the operational continuity of transport infrastructure. Among these hazards, potholes are particularly significant because they can cause tire damage, suspension wear, wheel misalignment, and sudden vehicle instability. In addition to direct mechanical damage, potholes may reduce driving comfort, increase maintenance costs, and degrade traffic efficiency in urban environments where roads are heavily used and rapidly deteriorate. For these reasons, the timely detection of potholes is an important requirement for road safety and infrastructure management. This work presents a tailor-made dataset for road pothole detection in urban environments, referred to as the Bridgestone Dataset (BS Dataset). The dataset was designed to support object detection from vehicle-mounted imagery collected from a test vehicle under realistic road conditions, thereby aligning the training data more closely with the target deployment scenario. The resulting dataset is intended to support real-time monitoring systems for road hazard detection and maintenance planning. The dataset was also designed as a multimodal resource. In addition to pothole bounding-box annotations, it provides accelerometer and GPS signals to characterize the vehicle dynamics during operation which might help identifying hazard severity and the potential risk to the vehicle. To collect the dataset, the authors developed a smartphone application, which supports the acquisition of both images and vehicle telemetry by leveraging the device’s internal sensors.

## 1. Introduction

Road infrastructure inspection is increasingly relevant in the context of intelligent transportation systems and smart mobility.

Within this field, pothole detection has received particular attention because potholes affect both vehicle operation and road maintenance. They may damage tires, reduce passenger comfort, and create unsafe driving conditions, especially when they are not detected in advance.

According to [1], pothole-related damage was responsible for approximately 207,000 vehicle repairs in Sweden in 2014, resulting in costs of nearly SEK 625 million. By 2019, these costs had increased to an estimated SEK 925 million, highlighting the growing impact of poor road conditions on motorists. In addition to vehicle repair expenses, Sweden faces substantial road maintenance challenges. The same source reports that prolonged underfunding has resulted in an estimated road maintenance backlog of SEK 25 billion. This backlog increased from SEK 16.5 billion in 2022 to SEK 19.1 billion in 2023 and, without additional investments, is projected to reach SEK 46 billion by 2033. As a consequence, up to 25% of the national road network could be classified as being in very poor condition, emphasizing the need for effective road monitoring and maintenance solutions.

The same authors reported a comparable situation across the United Kingdom and Europe [1]. The United Kingdom alone is estimated to have more than one million potholes, while many European countries are experiencing increasing maintenance backlogs as a result of aging infrastructure and insufficient funding. The consequences of road surface degradation are substantial, with UK motorists spending approximately £579 million per year on vehicle repairs associated with pothole damage, including tires, suspension systems, wheels, and other components. To address these issues, several governments have increased investments in road infrastructure; for example, the UK government allocated an additional £8 billion for road maintenance and pothole repairs in 2023. Nevertheless, repair activities continue to lag behind maintenance needs in many regions. At the European scale, the road maintenance backlog is estimated to amount to hundreds of billions of euros, and several countries report that a significant portion of their road networks requires renovation. These findings emphasize the growing economic burden of road deterioration and highlight the importance of developing effective road monitoring technologies and proactive maintenance strategies to improve road quality and safety.

The need for automated pothole monitoring is amplified in urban environments, where road usage is continuous and the consequences of infrastructure degradation may appear rapidly. In this context, the literature has increasingly emphasized real-time detection systems that can warn drivers, log road hazards, and support maintenance agencies [2].

Identifying hazards such as potholes is a challenging task for public infrastructure authorities due to limited resources and high costs related to continuous monitoring. In fact, most known approaches rely on visual inspections and public reports, which can be highly inefficient at scale and do not provide a quantitative assessment of road condition. More advanced approaches use dedicated vehicles and instruments with scanning technologies such as LiDAR and laser scanning, which can detect road defects with high precision [2]. However, such systems come with high costs and limited availability, making them impractical when applied at scale, especially for large infrastructures.

This has encouraged the adoption of fleet-based road infrastructure monitoring, where multiple vehicles are engaged to collect relevant data for inferring the current status of the road condition [3,4,5,6]. These approaches rely on monitoring large urban areas to quickly identify road hazards before they constitute a risk to road users, improving maintenance response times.

The growing availability of low-cost cameras, onboard sensing devices, and mobile computing platforms has encouraged the development of automatic road monitoring solutions [2].

At the same time, the problem remains challenging because pothole appearance is highly variable. Different road surfaces, lighting conditions, and acquisition viewpoints can affect visual cues. As a result, models trained on narrow datasets may not generalize reliably. This suggests that the availability of realistic, representative datasets is as important as the design of the detection algorithm itself.

In this work, we propose the Bridgestone Dataset (BS Dataset), a large road image dataset for pothole detection. It is built using a custom smartphone application called the PotholeByCamera App, which collects both visual data and sensor information during pothole-impact events. The data were collected during a public-road testing campaign in collaboration with the Bridgestone Europe NV/SA – Italian Branch, Technical Center Europe, located in Rome, Italy. The paper is organized as follows. First, we review existing methods for automatic pothole detection. Afterward, we describe the BS Dataset and the data collection process. Next, we present the experimental setup used to train different object detection models. Finally, we discuss the results and provide our conclusions.

## 2. Review of Pothole Detection Methods

This section provides a brief review of several state-of-the-art approaches for pothole detection available in the literature. Depending on the underlying technology, we distinguish between signal-based and image-based methods. We further divide image-based methods into traditional image-processing and deep-learning approaches.

### 2.1. Signal-Based Methods

Signal-based methods infer the presence of potholes from non-visual measurements, including vibrational (e.g., displacement, velocity, and acceleration) and acoustic responses. These systems are attractive because they can be implemented with relatively simple onboard sensors and may operate without direct image interpretation. In fleet-based scenarios, vehicles can collect telemetry data during routine travel, and the signals can be analysed to estimate the condition of the road surface [2].

Many authors use a combination of digital signal processing techniques with machine learning and deep learning algorithms applied to vehicle telemetry to detect when a vehicle impacts a pothole [3,4,5].

In contrast to most signal-based approaches, Alleva et al. propose, in their patent a method for pothole detection by analyzing wheel speed signals extracted from the vehicle Controller Area Network (CAN) bus [6]. Compared with conventional methods such as those based on vehicle acceleration, these high-frequency telemetry signals can be used to monitor the variation of the signal to detect tire impact events with road hazards such as bumps or potholes. These signals can support accurate estimation of geometric properties such as depth and length since they are more related to the vehicle’s tire properties rather than other vehicle characteristics, such as suspension and chassis. They describe a “P2P algorithm” defined as a combination of the wheel speed with the speed to accurately classify potholes with different heights and lengths.

Patent EP4330092A1 [7] presents a method for estimating the International Roughness Index (IRI) from connected-vehicle data collected during normal operation. The approach uses vehicle vertical acceleration, speed, geolocation, and tire characteristics to calibrate a predictive model capable of inferring road roughness without dedicated profiling equipment. A key feature is the Root Mean Square (RMS) of the vertical acceleration signal, which quantifies the overall vibration energy induced by the road surface and provides a robust measure of vehicle response by reducing sensitivity to transient peaks. By combining RMS acceleration with vehicle speed and tire parameters, the method establishes a relationship between vehicle dynamics and pavement roughness, enabling large-scale crowd-sourced road monitoring. However, the approach remains sensitive to vehicle-specific characteristics, such as suspension configuration, tire properties, mass, and loading conditions, which may limit its generalizability across different vehicle classes.

Although signal-based methods can be useful for road roughness estimation, they generally provide limited spatial precision. They may detect that an irregular event has occurred, but they do not always indicate the exact location or visual appearance of the pothole. Their performance can also depend on vehicle speed, suspension characteristics, and sensor placement. These factors also affect telemetry responses and therefore require accurate calibration. This requirement limits the adaptability of detection algorithms across vehicle configurations. Moreover, by definition they operate in a “reactive” way, meaning that they can detect a pothole only when an impact event occurs. For this reason many authors propose these systems to operate on multiple vehicles or fleets from a crowd-sensing perspective to maximize the probability that a vehicle drives over a road hazard [2,3,5], since drivers tend to avoid dangerous potholes by driving around them, consequently increasing the false-negative rate. These methods are also prone to false positives. Artificial road elements such as speed bumps, manholes, road intersections, and similar structures can incorrectly trigger the detection system. In addition, driving behavior can also cause false detections, especially during sudden acceleration, braking, or sharp turns.

### 2.2. Traditional Image Processing Methods

Traditional image-processing include methods that use handcrafted image features and rule-based pipelines. These methods often rely on edge detection, contour extraction, thresholding, or road appearance modelling. They are typically computationally efficient and easier to interpret than neural network-based approaches and can be implemented on edge hardware without strict computational requirements.

One of the earliest studies in this area was conducted by S. Nienaber et al. [8]. They proposed a system to detect potholes from images using a simple two-step method. The approach does not use machine learning, but instead relies only on basic image characteristics of road potholes. In the first step, the system identifies only the road area in front of the vehicle. This is done using color-based filters to find asphalt regions and a convex-hull algorithm to handle irregular shapes. In the second step, the system detects potholes using Canny edge detection, based on the idea that potholes usually appear in darker areas of the road. This method was tested on a dataset collected by the authors on public roads. This approach, while simple, is hard to apply to all types of images. Traditional image processing methods are sensitive to changes in illumination, color, camera position, lens characteristics, background content, and image noise. In addition, these techniques can incur high computational costs, which makes them difficult to run on edge devices, especially when working with high-resolution images or videos.

Another example is the work of Tedeschi et al. from the University of Roma 3. They developed a system called Automatic Pavement Distress Recognition (APDR), implemented as a smartphone application [9]. The application classifies different types of road damage, including fatigue cracks, longitudinal and transversal cracks, and potholes. The method uses LBP filters [10] to extract image features for image classification.

In his study, Nhat-Duc Hoang developed an image classification method for detecting potholes using Support Vector Machines (SVM) and artificial neural networks (ANN) [11]. He collected a dataset of road images divided into two groups: images with potholes and images without potholes. This dataset was used to train a system called the “Steerable Filter and Artificial Intelligence-Based Pothole Detection Model” (SF-AI-PDM). The system first applies image processing techniques: Gaussian filters to reduce noise, steerable filters to enhance the images, and integral projection to extract features. The extracted features were then analyzed using supervised learning methods, such as SVM [12] and neural networks, to determine whether an image contains a pothole or not.

Vision-based methods introduce a new workflow for pothole detection. Unlike signal-based methods, they enable proactive detection, because they do not require the vehicle to hit the pothole. Instead, they detect hazards using visual data alone. This approach is especially suitable for advanced driver-assistance systems (ADAS) and autonomous vehicles, where early detection can be used to adjust vehicle behavior or warn the driver through feedback systems. In addition, vision-based methods reduce the false-negative problem of signal-based approaches mentioned in the previous section, since triggering detection does not depend on a physical impact with the road surface.

Despite their strengths, traditional image processing pipelines often require careful parameter tuning. Changes in illumination, shadows, road markings, surface texture, or viewpoint can reduce detection reliability. Consequently, such methods may be less suitable for deployment in highly variable urban scenes unless they are supported by strong acquisition control. Achieving real-time detection also involves computational trade-offs. Running these algorithms can be computationally expensive, especially when they process large images and require multiple processing stages.

### 2.3. Neural-Network-Based Image Processing

More recent studies for road distress analysis have shifted toward neural network-based image analysis [13]. These methods have proved highly effective for numerous image-related tasks reported in the literature following advances in deep convolutional neural networks (CNNs).

Existing pothole detection approaches can be broadly categorized into three groups: classification, segmentation, and object detection. Classification methods determine the presence or absence of a pothole within an image, segmentation methods provide pixel-level delineation of road defects, and object detection methods identify and localize one or more potholes through bounding boxes.

Among these approaches, object detection has become a core task in computer vision, with applications ranging from image understanding and human behavior analysis to face recognition and autonomous driving. The advent of deep learning has significantly advanced object detection by overcoming the limitations of traditional methods, which relied on manually engineered features and descriptors. Instead, deep learning models automatically learn hierarchical feature representations directly from data, enabling greater robustness, expressiveness, and detection accuracy in complex real-world scenarios [14].

These methods can be adapted to road-safety use cases such as detecting speed bumps, potholes, and other asphalt anomalies [13,15,16,17,18,19].

A common distinction in object detection is between one-stage and two-stage detection architectures.

Two-stage models, such as those in the Region-Based Convolutional Neural Network (R-CNN) family (like R-CNN, Fast R-CNN, and Faster R-CNN), were among the first models to achieve strong performance on object detection benchmarks [20,21]. These models act in two steps: first, they generate region proposals that are likely to contain objects, and then they classify and refine these proposals. This process requires multiple components that may require separate training, thus increasing the computational cost and time required for inference.

One-stage object detectors such as You Only Look Once (YOLO), Single Shot MultiBox Detector (SSD) and RetinaNet are designed for speed and efficiency by performing both object localization (bounding box regression) and classification (object classification) in a single forward pass of the network [22,23,24,25]. These unified architectures significantly reduce computational costs, making them much faster for both training and inference. Overall, one-stage detectors offer a practical balance between speed and accuracy, especially when real-time performance is required, while two-stage detectors have a more modular architecture which guarantees higher performance and flexibility but with a much higher computational cost.

Despite the advantages of vision-based detection methods, the lack of a physical representation allows only the detection of the obstacle, reducing the possibility of assessing hazard geometry and, consequently, the associated risks to the vehicle. In their review of road anomaly detection methods Rohit et al. recognized that academic AI models often excel on curated datasets but struggle in real-world deployment due to domain shift, limited generalizability, and unrealistic assumptions [13]. According to their research, a compelling future scope involves a holistic, research-driven roadmap leveraging advanced artificial intelligence, federated learning, and real-time sensor fusion for road anomaly detection and predictive safety interventions. One method to bridge the gap between research and real-world deployment is to integrate multi-modal data (vision, vibration, and geospatial) and adopt smart crowd-sourcing architectures to create adaptive, collaborative vehicle systems capable of dynamic risk prediction and coordinated response.

To address these challenges, we investigated a hybrid approach that integrates the visual analysis capabilities of deep learning models with signals generated by pothole-related events. The proposed framework is designed not only to detect road surface anomalies but also to estimate their severity, thereby providing a comprehensive assessment of road conditions. Furthermore, by leveraging complementary data sources, the system enables the proactive identification of potholes before they pose significant risks to road infrastructure and road users.

## 3. Pothole Detection Datasets

The availability of suitable datasets strongly affects performance in image-based road-distress research. Existing datasets vary widely in size, data collection conditions, labeling methods, and how well they represent real-world scenarios. Since this work focuses on pothole detection, we review previous studies that specifically address this type of damage.

### 3.1. Public Sources

In their review, Rohit et al. examined the current state of datasets used for road-anomaly detection [13]. They analyzed several state-of-the-art datasets designed for this task and pointed out key limitations and challenges in selecting suitable datasets for deep learning approaches:

1. Data quality and acquisition conditions: visibility, lighting, weather, blur, camera quality, and sensor placement are major factors that can introduce noisy or unclear images that reduce detection reliability;
2. Label quality and reliability: poor annotation standards and inconsistent anomaly definition create dataset biases in detection tasks, especially when trying to combine multiple sources, also some authors used automatic annotations techniques which lower the annotation reliability;
3. Class representation and imbalance: many sources suffer from severe class imbalance across several categories, particularly potholes, which are underrepresented;
4. Geographic and domain diversity: region-specific datasets can introduce training bias and increase the risk of domain shift.

**Figure 1:**
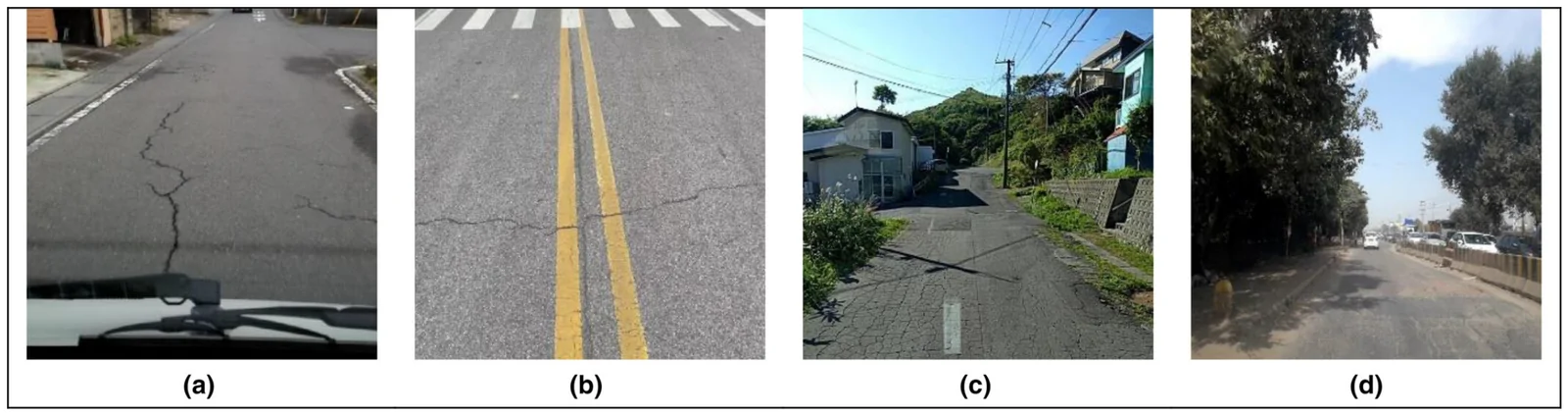
Different road damage categories considered in the 2022 Road Damage Dataset [19]. (**a**) Longitudinal Crack (D00), (**b**) Transverse Crack (D10), (**c**) Alligator Crack (D20), (**d**) Pothole (D40).

According to these criteria, the authors identified RDD2022 [19] as the most valuable dataset for general road-anomaly research. The RDD2022 is a large, multinational dataset designed to support automatic detection and classification of road damage using deep learning. It includes 47,420 road images collected from six countries and provides annotations for more than 55,000 road damage instances. The damage is grouped into four main categories as shown in Figure 1: longitudinal cracks, transverse cracks, alligator cracks, and potholes. The dataset was created for the Crowdsensing-based Road Damage Detection Challenge (CRDDC-2022) [26], which aimed to develop models that perform well across different countries. The images were captured using various devices, such as smartphones, cameras, and Google Street View, and collected from different vehicles, including cars, motorcycles, and drones. Despite its overall diversity, the pothole category is one of the least represented in the dataset, with only 1888 images containing potholes.

Another dataset included in our study is the one proposed by S. Nienaber et al. [8].

The dataset consists of high-resolution images captured at regular time intervals using a GoPro Hero 3+ mounted on a vehicle’s windscreen. The images were divided into two groups. Images containing potholes were labeled as positive, while images without potholes were labeled as negative. For the positive images, bounding boxes were added to accurately show the location of the potholes in the image. The authors also provided two variants: a simple subset and a more complex subset, based on scenery complexity, visibility, foliage content, and other scene characteristics.

The last dataset we decided to include was the dataset introduced by Chitale et al. [15]. This is a collection of images obtained from the Google search engine featuring roads with potholes. Unlike the other datasets, the images in this one are highly varied, with differences in size, shape, camera position, and other characteristics. Nevertheless, this dataset proved to be effective in many studies and its format is similar to the Microsoft COCO dataset [27], which makes it ideal for fine-tuning object detection algorithms.

Table 1 summarizes the public datasets considered for the pothole detection task.

### 3.2. Proposed BS Dataset

The Bridgestone Dataset (which we will refer to as the BS Dataset) was created to support pothole detection in realistic urban driving environments. Its purpose is to provide a tailored image collection for object detection under conditions that resemble those encountered by a vehicle in motion. Differently from multi-class cases, a task-specific dataset is valuable because it can reduce the mismatch between training and deployment conditions, focusing on a specific application. In the present case, the BS Dataset is intended to capture road scenes that are visually relevant to real driving, including the effects of motion, angle variation, and changing road texture. This makes it particularly appropriate for evaluating real-time road hazard monitoring systems. To meet these criteria, the proposed BS Dataset was designed with the following characteristics:

- Vehicle driving context: images acquired from an onboard vehicle to match in-vehicle applications;
- High image quality: enables the detection of small objects;
- Scene complexity: high scene diversity, comprising urban, suburban, and rural environments, including complex traffic conditions;
- Large image/box count: to support the training of deep-learning algorithms on large datasets;
- Multimodality: includes vehicle telemetry signals to support hazard assessment.

The Bridgestone Dataset (BS Dataset) is a proprietary dataset owned by Bridgestone NV/SA, Italian Branch Technical Center Europe, and is therefore not intended for public release. The dataset may contain potentially sensitive information collected in real-world driving environments, including images of public roads, pedestrians, vehicles, license plates, buildings, and other identifiable elements. For this reason, access to the BS Dataset is restricted and subject to appropriate confidentiality and data protection requirements. Researchers interested in accessing the dataset may contact the authors to discuss potential collaboration opportunities and data-sharing agreements.

#### 3.2.1. Construction: PotholeByCamera App and Hybrid Data Strategy

The primary goal of dataset acquisition was to collect images of road potholes along with sensor information to estimate hazard severity and vehicle location. As a second goal, we aim to keep the system low-cost and easy to install, promoting compatibility across different devices and vehicles. For these reasons, we therefore developed an Android smartphone application. Current smartphones come equipped with all the key sensors needed for this task, including high-quality cameras, location capabilities through GPS and built-in IMU (Inertial Measurement Unit) sensors like accelerometers and gyroscopes. They also provide high computational power and storage, along with long-lasting batteries that could support several hours of operation. Another important factor is that Android devices are relatively easy to program and customize, making development faster and more flexible. Compared to industrial-grade cameras and sensor systems, smartphones are also much more affordable, which makes them a cost-effective choice for building and scaling our solution. The data collection application covers three main functionalities:

1. Collect high-resolution images of potholes;
2. Acquire telemetry signals related to vehicle dynamics during pothole-impact events;
3. Locate the vehicle position and speed using GPS services.

The main challenge of the application is to be retroactive in the data acquisition, since we want to store the images of the obstacle before the collision and the signals during the event. To do so, we designed the system to continuously record a video until a road hazard event is detected. Events are detected by applying predefined thresholds to signals from the smartphone’s built-in sensors.

For the sensing capabilities Table 2 lists the Android API sensor measurements used by the application.

We include two types of accelerometer sensors: accelerometer and linear-acceleration, both sampled at the base frequency of 50 Hz. The first is hardware-based and measures only the acceleration of the device, while the second combines the accelerometer with other sensors such as the magnetometer and gyroscope to improve the signal accuracy and to remove the gravitational component.

In order to quantify the vehicle acceleration induced by the collision with potholes we implemented a simple RMS calculation of the 3-axis acceleration of the device:

(1) $$RMS_{Accel}=\sqrt{\frac{1}{N}\sum_{i}^{N}\left(a_{x}^{2}+a_{y}^{2}+a_{z}^{2}\right)}$$

where $a_{x},\;a_{y},\;a_{z}$ are the acceleration components in the three spatial directions in m/s^2^. Based on this formula we implemented two resampled versions of the RMS to be used in the application with thresholds in order to detect collision events with road potholes.

- Sensitivity 10 Hz: accelerometer RMS threshold computed at 10 Hz;
- Sensitivity 1 Hz: accelerometer RMS threshold computed at 1 Hz.

Using two thresholds allows the system to detect different types of collisions: the first targets short events caused by a single pothole impact (10 Hz), while the second can identify longer events related to roads in poor condition with multiple defects (1 Hz). During operation, the application continuously computes the RMS value. When the vehicle hits a pothole, the system compares the signal magnitude at both frequencies with predefined thresholds. If the signal exceeds a threshold, the system saves a video of the event in the device memory together with all related telemetry data.

For localization we included GPS logging, in order to localize identified potholes and estimate the vehicle speed during the event.

For the vision component of the application, we implemented the camera functionality using the *CameraX* API by developing a video-capture feature. This allows the system to record high-resolution videos with low compression, from which individual frames can be extracted for data collection.

**Figure 2:**
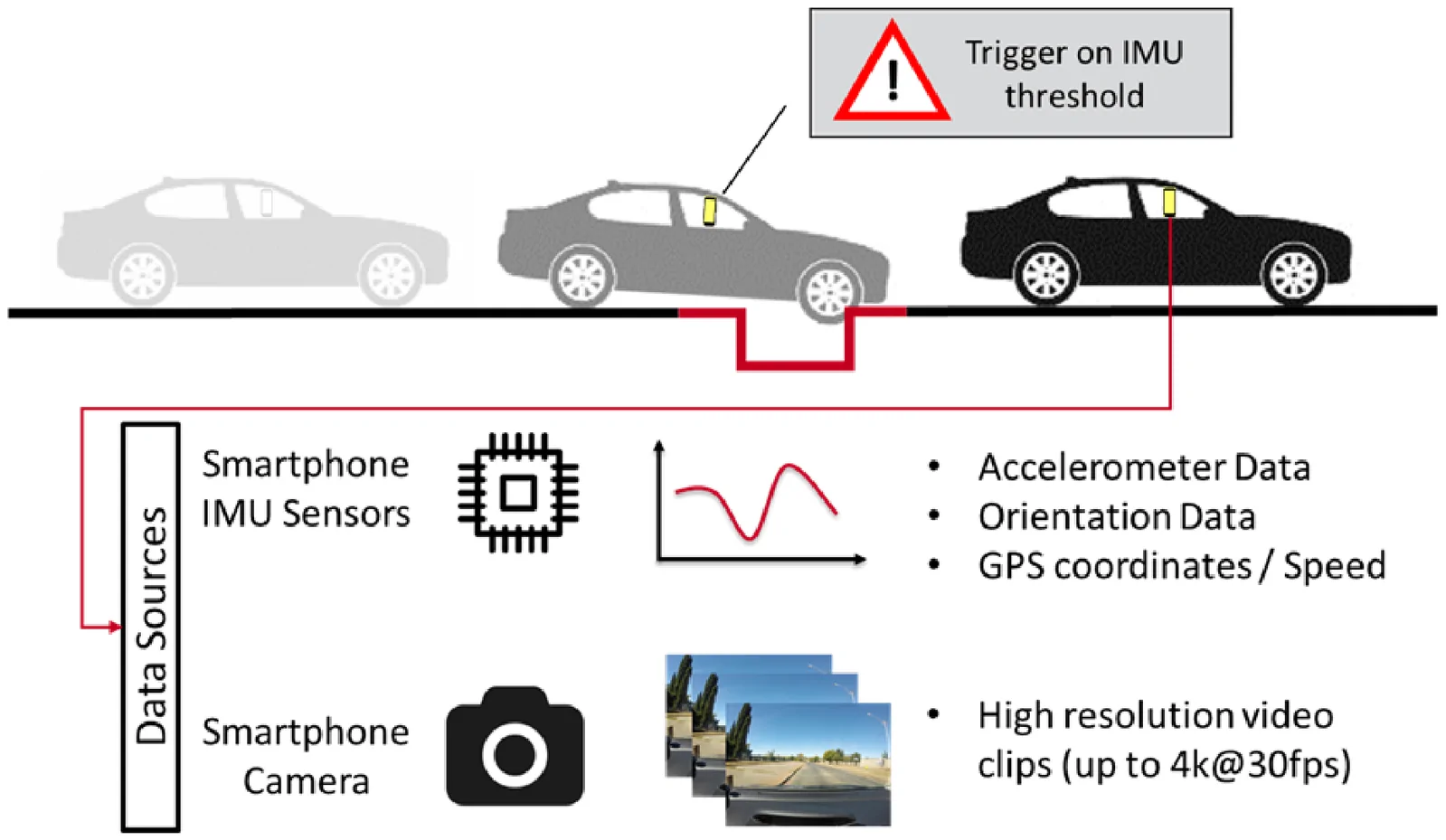
Data acquisition flow of the PotholeByCamera application. A vehicle is driving on the road with a smartphone running the application. When a collision with an obstacle on the road occurs the built-in sensors of the device trigger the application data collection function. The smartphone saves the video session along with sensor signals and GPS data.

Figure 2 summarizes the data-collection use case. In this setup, a vehicle travels with a smartphone running the data acquisition system. When the vehicle hits a pothole, the device’s built-in sensors detect the event and trigger a function that retrieves the video frames recorded before the impact, along with sensor data, vehicle location, and speed.

#### 3.2.2. Data Collection

After testing different configurations, we decided to mount the smartphone on the windscreen of a Volkswagen Golf 7 test vehicle using a suction cup phone holder. The support has a simple design where the base is made of an adhesive rubber compound that is attached to the vehicle windshield. A lever at the base of the cup creates a vacuum which firmly secures the piece to the glass. An extendable arm is connected to the cup by a rotating joint, allowing different inclinations. Finally, the arm is connected to the smartphone holder through a ball head with a screw locking mechanism to set the preferred angle of the device.

**Figure 3:**
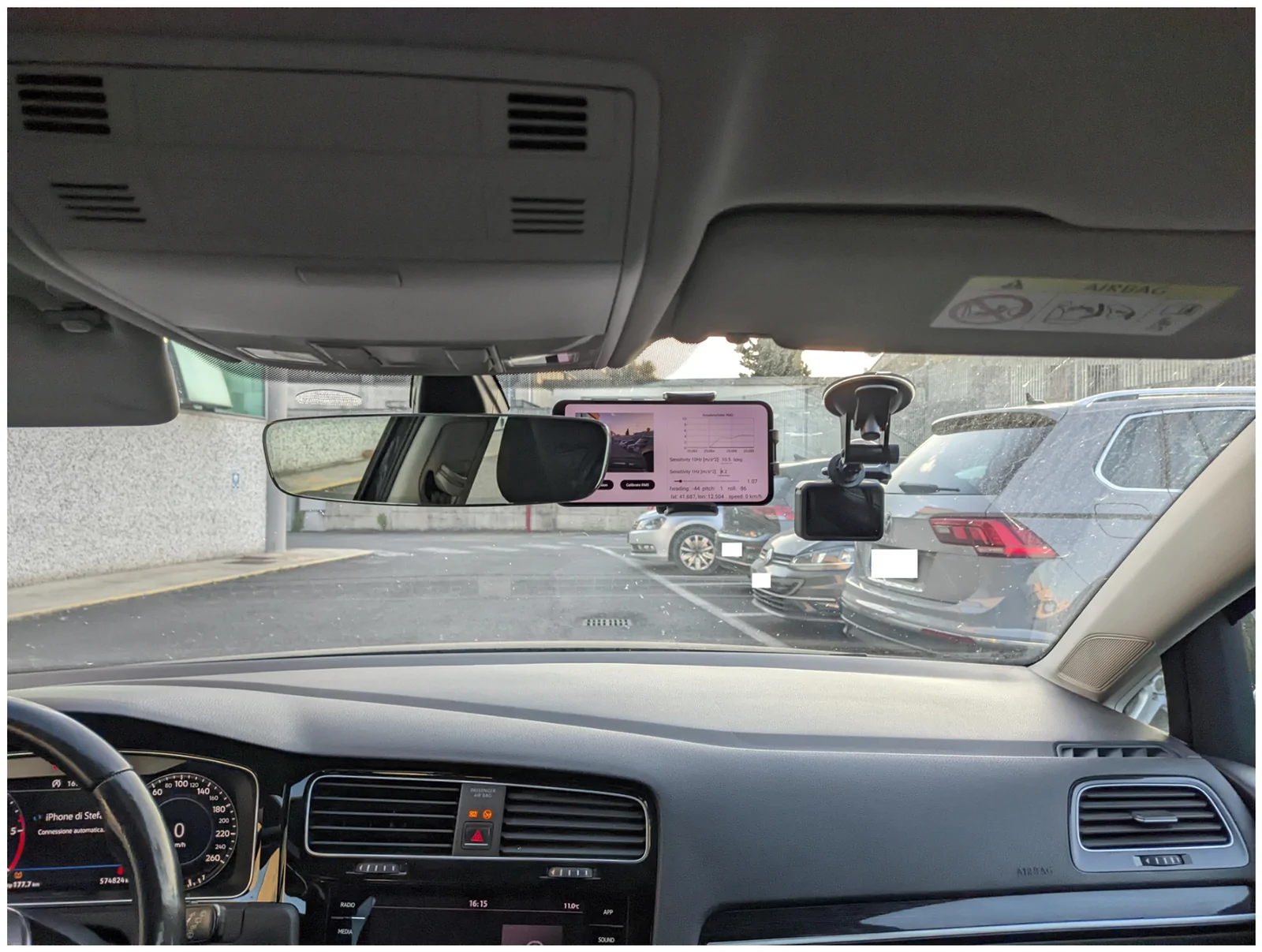
Smartphone setup for the test vehicle, a Volkswagen Golf 7. The smartphone is anchored to the vehicle windscreen using a suction cup-based phone mount.

The arm was positioned in the highest point of the windscreen, to increase the camera field of view in front of the car while reducing view obstruction. The arm length was minimized to reduce reflections and leverage vibration of the smartphone. Figure 3 shows the mounting setup of the smartphone system on the testing vehicle a Volkswagen Golf 7.

We tested the application on a variety of smartphone devices from different brands including Samsung, Xiaomi and Motorola. On all tested devices the *linear_acceleration* software-based sensor proved to be the most stable and repeatable; however, it is not supported by every device since it requires a gyroscope. For this study, we primarily used the Samsung Galaxy A55, which provides a good compromise of image/signal quality while being affordable and reliable for long testing sessions. The main hardware specifications of the Android smartphone are reported in Table 3.

The images were collected on public roads, so they reflect real driving conditions, including the typical viewpoint and motion of a moving vehicle. Compared to static or web-collected images, this approach introduces realistic challenges such as oblique angles, partial occlusion, motion blur and dynamic lighting conditions, which are important for real-world pothole detection. The dataset was collected between 2023 and 2024 and includes 17,553 road images and more than 24,704 pothole instances from the Italian regions of Lazio, Marche, and Umbria.

**Figure 4:**
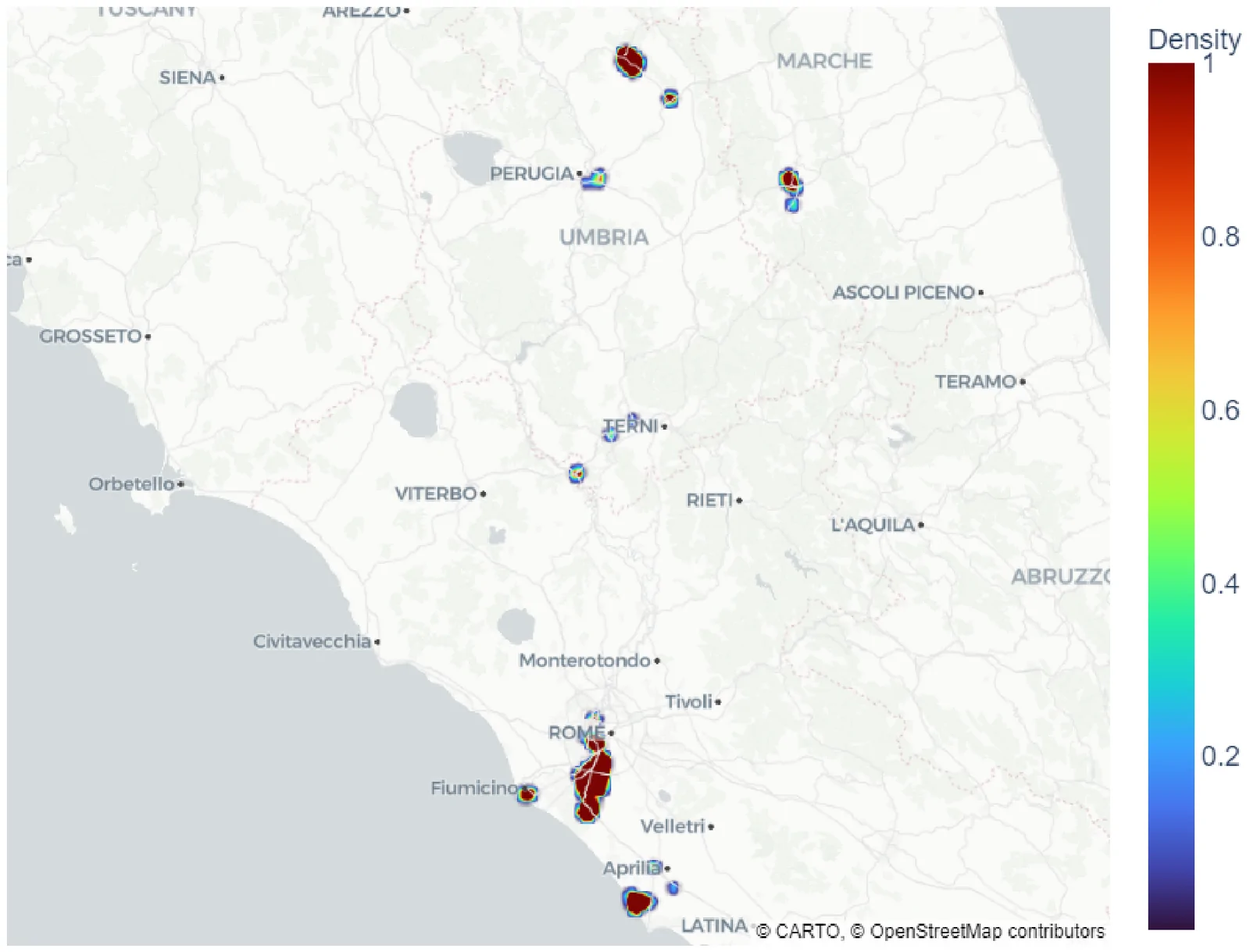
Dataset spatial density map.

Figure 4 presents the GPS density map of the collected dataset.

Of these images, 5250 also contain valid vehicle telemetry data recorded during collisions with potholes, with a total of 8142 annotated bounding boxes.

#### 3.2.3. Annotation Method

**Figure 5:**
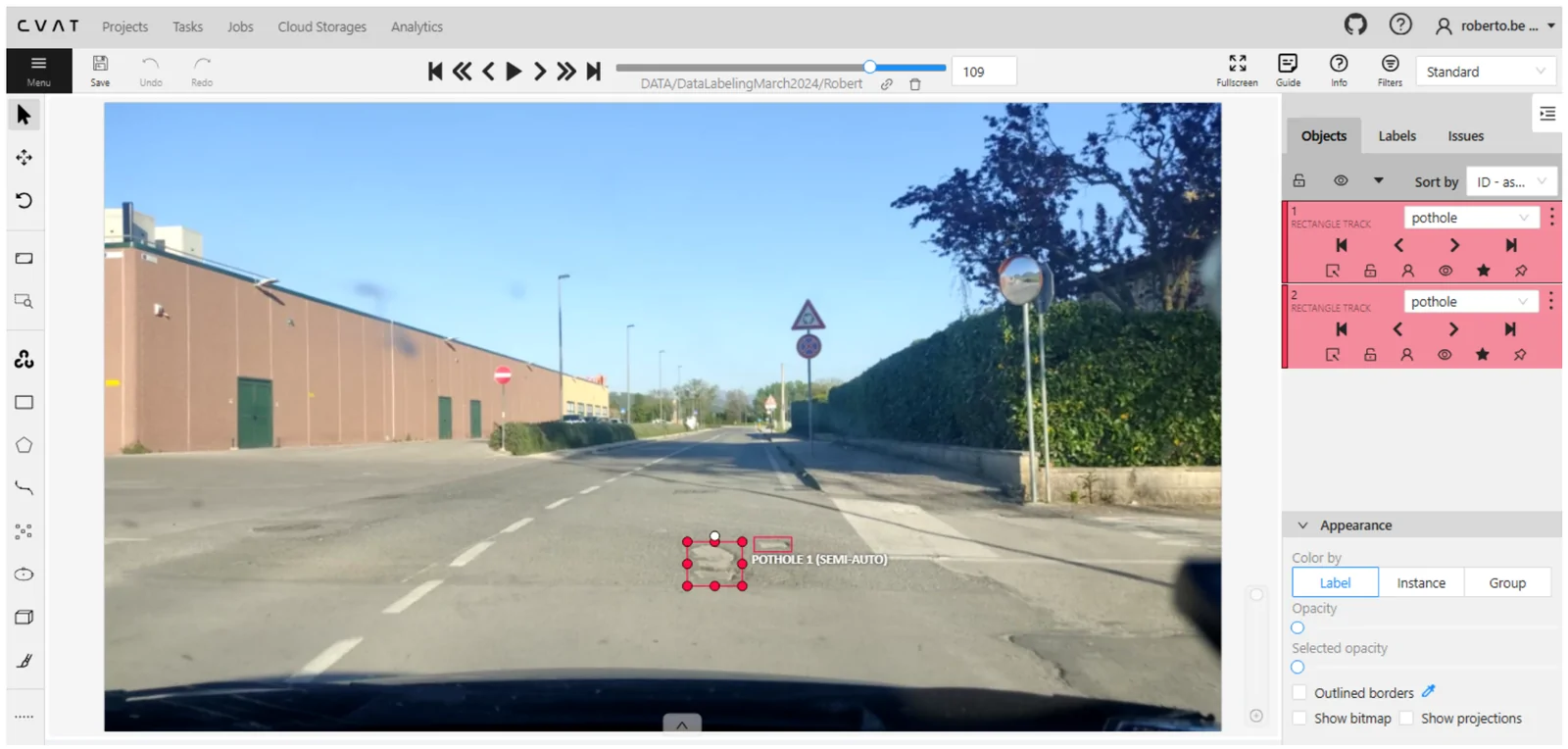
An example of pothole labeling using the CVAT tool on video. Through the graphical user interface, users can easily annotate individual video frames by tracking objects using computer-assisted tools.

The labeling process is critical for the performance of deep learning algorithms. Since the model needs to learn from many examples, thousands of images must be labeled accurately. Creating box labels requires considerable time and effort, especially when aiming to produce a high-quality dataset. To address this task efficiently, we chose to use a private instance of the Computer Vision Annotation Tool (CVAT) developed by Microsoft. CVAT is a web-based application with a user-friendly graphical interface that offers many tools designed to help with image annotation. Using this tool accelerated the labeling process and improve the quality of the annotations. One important feature is that it supports frame-by-frame video annotation. Figure 5 illustrates a video annotation instance in the CVAT user interface.

The annotation process was carried out by a team of five annotators, including the two authors, following a set of shared guidelines to ensure consistency across the dataset. Annotations were restricted to road potholes, while other pavement features and defects, such as cracks, pavement joints, curbs, road markings, and surface irregularities not corresponding to potholes, were intentionally excluded. Bounding boxes were generated using a standard minimum target size of approximately 150 × 70 pixels at the native image resolution of 3840 × 2160 pixels, ensuring the reliable labeling of clearly visible defects. To improve annotation quality, all labels underwent mutual review among annotators, with particular attention given to ambiguous cases. Discrepancies and uncertain annotations were discussed collectively until a consensus was reached, resulting in a more consistent and reliable ground-truth dataset.

For the telemetry data, we reviewed the application logs both manually and automatically, to remove false-positive events and outliers. These false detections were often caused by vehicle dynamics such as cornering and braking, or by non-pothole road elements like manholes, speed bumps, and road intersections.

## 4. Experimentation

The experimentation section of this research paper is dedicated to evaluating and comparing the performance of different object detection models. The main purpose of this part is to analyze how various detection approaches perform under the same conditions and dataset. To provide a comprehensive comparison, we included multiple architectures that represent both one-stage detectors and two-stage detectors. This allows us to study the advantages and limitations of each approach in terms of detection accuracy, speed, and overall performance.

Although the dataset includes additional vehicle telemetry signals, such as speed, acceleration, and geolocation information, the integration of these modalities is beyond the scope of the present study. Future work will investigate multimodal sensor fusion approaches that combine visual information with vehicle telemetry data to improve detection robustness, contextual awareness, and road condition assessment under diverse operating conditions.

All experiments were performed on a workstation featuring an Intel Xeon Gold 6148 CPU, 384 GB of RAM, and two NVIDIA Tesla V100 GPUs with 32 GB of VRAM each. The software environment consisted of Python 3.9.16, PyTorch (version 2.6.0), TorchVision (version 0.21.0), NumPy (version 1.26.4), Ultralytics (version 8.4.50) and CUDA (version 12.4) for GPU-accelerated computation.

The chosen model lineup is reported in Table 4.

The first algorithm in our comparison is the Faster R-CNN model [21]. With an input resolution of 800 × 800 pixels, it is a two-stage object detector that combines a ResNet50 backbone with a Feature Pyramid Network to extract multi-scale features, and it uses a region proposal network followed by a classification and regression head in a PyTorch-based implementation optimized for balanced accuracy and robustness.

The second algorithm is the one-stage object detector RetinaNet [23] with an input size of 800 × 800 pixels. It also employs a ResNet50 backbone with an FPN but introduces focal loss to address class imbalance, and its implementation focuses on stable dense prediction with improved detection of small and hard objects.

Next we included some variants of the YOLO family, starting from the YOLOv5mu [28] model with 640 × 640 pixels input. This is the medium-sized one-stage detector using a CSP-based backbone and path aggregation neck, with an efficient PyTorch implementation designed for real-time inference and a good trade-off between speed and accuracy. The YOLOv5m6u [28] model extends the base architecture to a higher input resolution of 1280 × 1280 pixels by modifying the stride and anchor scales, leading to improved detection of small objects at the cost of increased computation and memory usage.

The YOLO26m [29] model with 640 × 640 pixels input is a larger and more expressive variant that increases the model capacity and depth without using NMS and anchor boxes, aiming to achieve higher accuracy in complex scenes while still following a single-stage detection pipeline optimized for GPU and edge deployment.

Finally, we also evaluated the Real-Time Detection Transformer (RT-DETR) model [30]. Using a 1024 × 1024 pixels input resolution, RT-DETR is a real-time transformer-based detector that replaces traditional anchor-based methods with a set-based global matching strategy. The model relies on an efficient encoder–decoder transformer architecture, allowing it to achieve competitive detection accuracy while maintaining fast inference speed.

### 4.1. Dataset Preparation

Because we are working with multiple data sources, we decided to create separate splits for each data source to maintain the same proportion of each subset and to evaluate their individual performance. For the public datasets, we decided for a randomized train-validation-test with proportions 70%, 20% and 10%.

For our custom dataset collected through road testing, we add two extra steps to the data-splitting process: downsampling and stratified sampling. The downsampling step reduces the number of training images by lowering the video framerate, since consecutive video frames are highly correlated and could cause overfitting during training. The stratified sampling step groups frames belonging to the same timestamp second before splitting, to prevent very similar images from appearing in different sets, which could lead to training contamination.

To thoroughly test training and model generalization, we also created two additional combined datasets: “all public”, which merges the three public datasets, and “all datasets”, which includes both the public datasets and our BS Dataset.

Table 5 summarizes the datasets counts for the three splits.

#### 4.1.1. Data Augmentation

Data augmentation is a method used to increase the variety of a training dataset by applying simple changes to existing images, such as rotation, flipping, scaling, or changing colors. It is useful when training deep neural networks because it helps the model see data in different forms and hence increases generalization. This means the model can learn better and perform well on new images it has not seen before. In our experiments, we applied data augmentation during training by using random transformations on each batch in real time with the torchvision library. This means that at every iteration, the input images are slightly changed in different ways before being passed to the model.

The augmentation techniques included in the training procedure of our models are

- RandomIoUCrop: crops a random portion of the image by ensuring that the new boxes satisfy a certain IoU with the original boxes;
- RandomHorizontalFlip: mirrors the image along the horizontal axis;
- RandomRotation: rotates the image by a random angle;
- ColorJitter: alters the image brightness, contrast, saturation and hue;
- GaussianBlur: applies a Gaussian blur filter.

**Figure 6:**
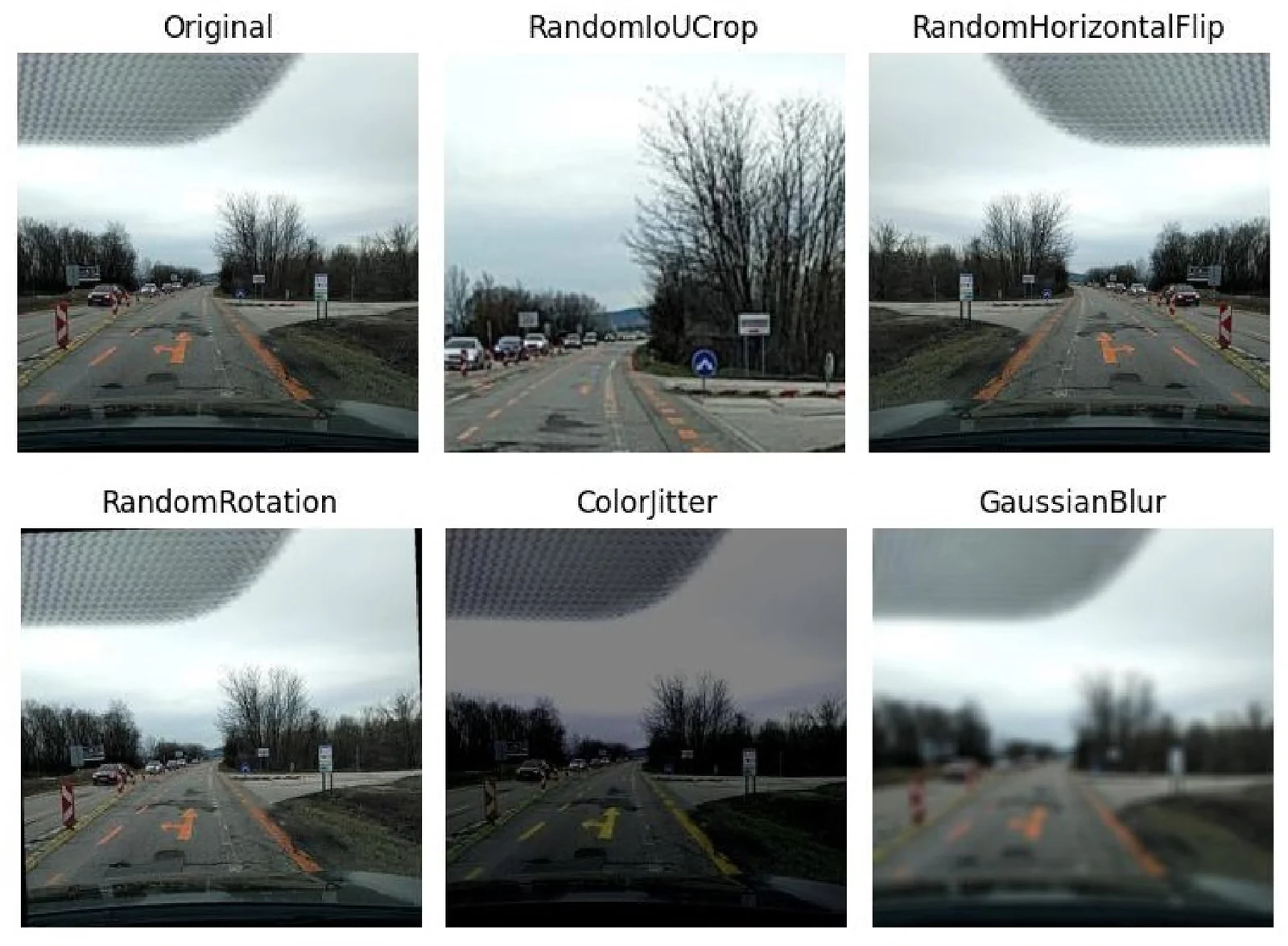
Examples of real-time augmentation techniques used in the model training pipeline.

Figure 6 illustrates different augmentation techniques used in this work.

#### 4.1.2. Evaluation Metrics

The evaluation of object detection methods differs significantly from standard classification tasks. In a traditional classification problem, each dataset sample is associated with a single label, which may belong to a binary or multi-class category. Model evaluation is therefore performed by directly comparing the predicted label with the ground-truth label. A True Positive (TP) occurs when a positive label is correctly identified, while a True Negative (TN) corresponds to the correct prediction of a negative label. A False Positive (FP) is produced when the model incorrectly predicts a positive label, whereas a False Negative (FN) occurs when the model fails to detect a positive instance.

**Figure 7:**
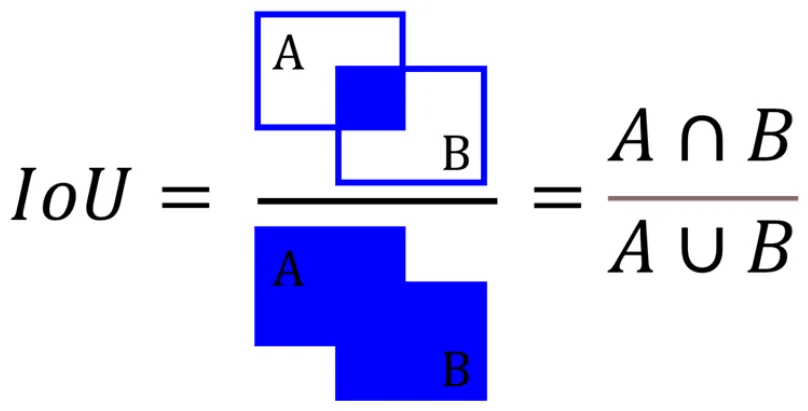
Object detection metric: Intersection over Union (IoU). The formula evaluates the predicted box by computing the ratio of the intersection area with the ground-truth box over the area of the union of the two boxes.

In object detection, however, a single image may contain multiple objects belonging to different classes. Each object is represented by a bounding box, defined as a set of coordinates that delimit the object location within the image. As a result, evaluation requires not only the correct classification of each object, but also the accurate localization of its position. This is achieved by comparing a ground-truth bounding box $b_{g}$ with a predicted bounding box $b_{p}$ through the Intersection over Union (IoU) metric, which measures the ratio between the overlapping area of the two boxes and the area of their union. Figure 7 illustrates the Intersection over Union (IoU) metric.

If the IoU between $b_{g}$ and $b_{p}$ exceeds a predefined threshold, commonly set to IoU ≥ 0.5, and the predicted class matches the ground-truth label, the detected object is considered a True Positive (TP). Otherwise, the prediction is classified as a False Positive (FP). In object detection tasks, negative instances are generally associated with the image background or with regions that do not contain any object of interest. Since a single image may include a very large number of background regions, the number of True Negatives (TN) becomes considerably high and less informative for evaluating model performance. For this reason, object detection metrics mainly focus on the balance between correct detections and localization errors rather than quantities based on True Negative counts.

Given a confidence threshold for the classification and an IoU threshold for the boxes, we can compute the Precision, Recall and F1 score just as for the classification task:

(2) $$Precision=\frac{TP}{TP+FP}$$

(3) $$Recall=\frac{TP}{TP+FN}$$

(4) $$F1=2\cdot \frac{Precision\cdot Recall}{Precision+Recall}$$

To ensure reproducibility, we employed the evaluation procedure implemented in the Ultralytics package [29], which provides F1-maximized Precision and Recall scores by varying the confidence parameter.

One of the most widely used evaluation metrics in object detection is the Average Precision (AP). By changing the confidence threshold of the detector, different Precision and Recall values can be obtained, producing a precision–recall curve for each object category. The Average Precision is defined as the area under this precision–recall curve and provides a measure of the detector’s performance by combining both localization accuracy and classification capability into a single metric.

(5) $$AP=\int_{0}^{1}Precision\left(Recall\right)\;d\left(Recall\right)$$

Since object detection is defined as a multi-class problem, we can also define the Mean Average Precision (mAP) which is the mean AP of all classes:

(6) $$mAP=\frac{1}{N}\sum_{i=1}^{N}AP_{i}$$

where *N* is the number of class labels and $AP_{i}$ is the Average Precision for the ith class.

A further extension of the mean Average Precision (mAP) metric involves computing it across multiple IoU thresholds. For example, evaluating mAP at an IoU threshold greater than 50% yields the mAP@50 score. A more comprehensive evaluation is obtained by averaging the AP values over a range of IoU thresholds from 0.5 to 0.95 with a step size of 0.05, resulting in the mAP@0.5:0.95 metric. This provides a more robust measure of detection performance by accounting for both classification accuracy and localization precision across varying levels of predicted box confidences.

## 5. Discussion: Experimental Results

In this section, we present the experiments conducted on different pothole datasets including both public datasets and our proposed BS Dataset Table 5. During the testing phase, we trained and evaluated all the models across different dataset combinations to assess model accuracy, training effectiveness, and generalization performance. The results are reported in Table 6.

A first observation can be made from the results obtained when training on individual datasets. In general, models trained on a single dataset perform well on the corresponding test set but show poor results when evaluated on other datasets. This limitation is likely due to domain shift, since each dataset contains significant differences in data distribution, which reduces generalization and limits the effectiveness of cross-domain learning. Among the datasets, RDD2022 shows the best overall performance due to its more diverse sources and geographic regions. However, it still does not provide enough variability to ensure strong generalization across all datasets, whereas the best-performing model, RT-DETR-L, achieved a mAP@50 of 0.288 on the full test set.

Different observations can be made for the models trained on the combination of public datasets (all public). This training setup not only improves overall performance across different test sets, but in some cases also surpasses the results obtained when training and testing on the same dataset. For instance, YOLO26m trained and tested on the Chitale et al. dataset achieves an F1 score of 0.571, while the same model trained on the public dataset mixture reaches a higher F1 score of 0.675. This indicates that, despite the increased variability and potential domain shift, using a larger and more diverse training set generally leads to better results by improving model generalization and training stability.

However, even with these improvements, models trained on the public ensemble still show limitations on the BS Dataset, where the best-performing model, RT-DETR-L, achieves a mAP@50 of 0.347 and an F1 score of only 0.426 on the BS Dataset test set. This performance drop can still be attributed to domain shift, as the BS Dataset consists of video-based frames that are affected by motion blur and compression artifacts, which are less present in single-image datasets. In addition, more complex scene conditions such as traffic, vegetation, and shadows further increase the likelihood of false positives.

Regarding the training results on the BS Dataset, we observe similarly weak performance when models are tested on other public sources. On the other hand, combining the public datasets with the BS Dataset (all datasets) leads to the best overall results. In this setting, RT-DETR-L is the best-performing model of the comparative study, achieving the highest metrics across all test sets when trained on the full dataset (mAP@50 score of 0.658 and an F1 score of 0.684 on the global test set). We believe that including public sources helps regularize the training process by increasing data diversity and improving overall generalization.

**Figure 8:**
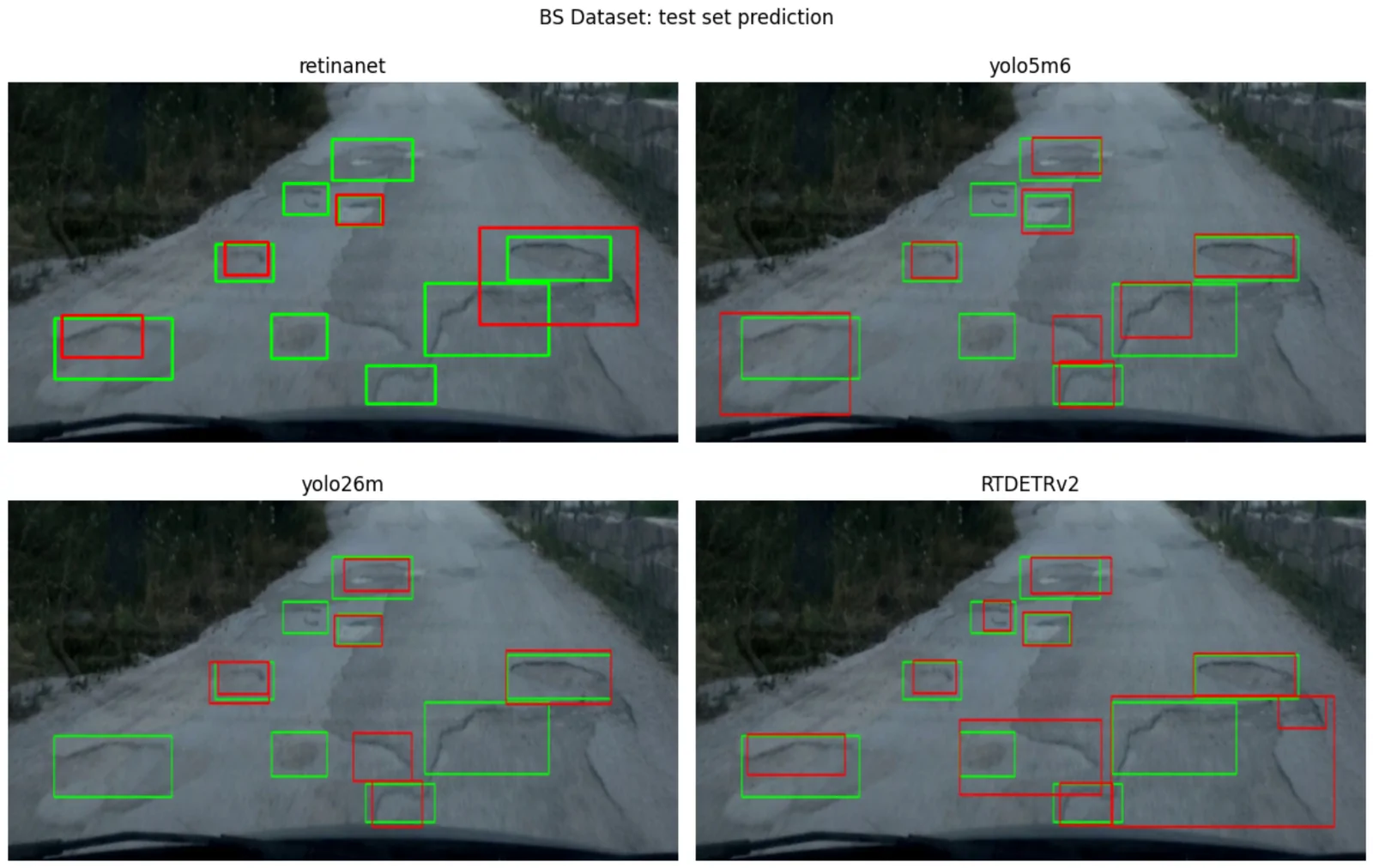
Prediction comparison between different models on the BS dataset. Green boxes represent ground-truth objects, while red boxes represent model predictions.

**Figure 9:**
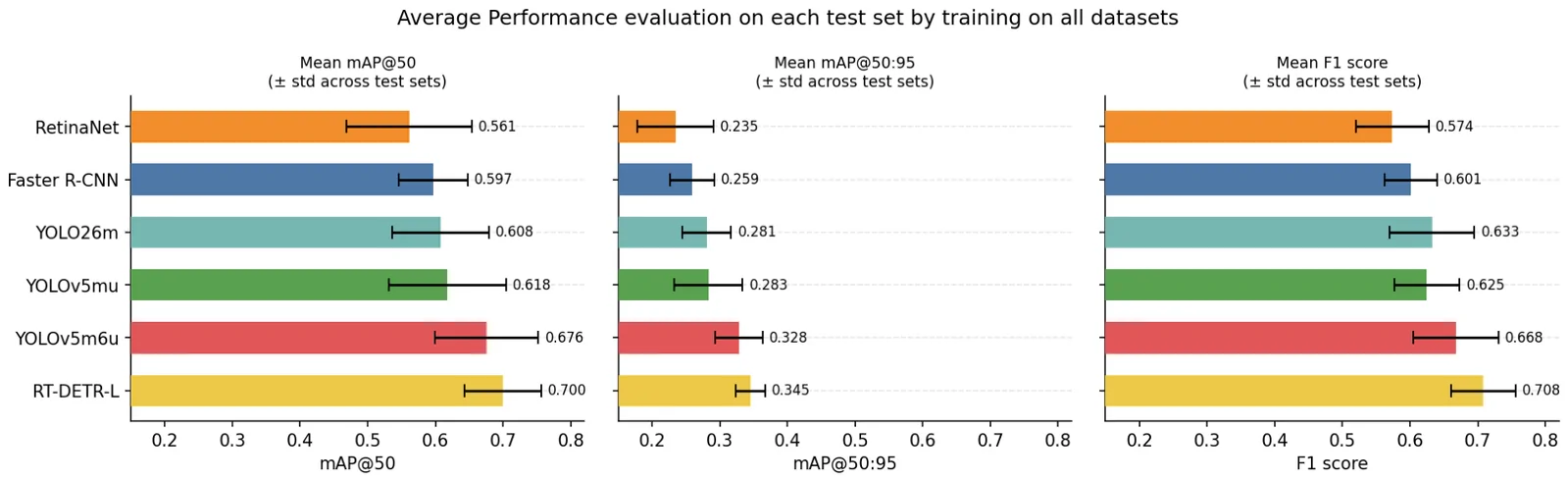
Performance of all models on each test set after training on the combination of all training sets. Each bar shows the average result for a dataset, and the error bars show the standard deviation.

**Figure 10:**
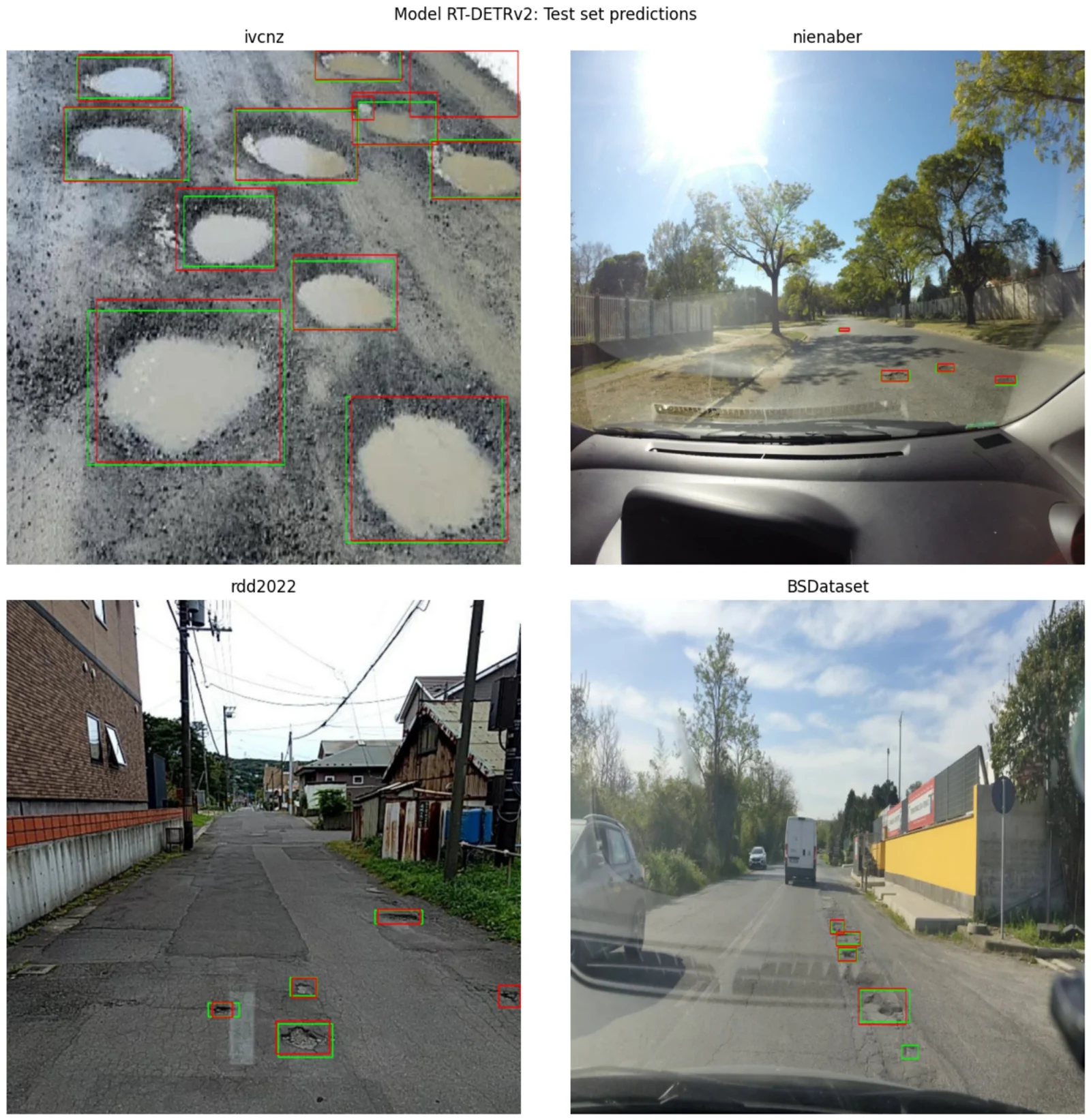
Test set predictions produced by the RT-DETR-L model. Green bounding boxes indicate the ground truth annotations, while red bounding boxes represent the model predictions.

**Figure 11:**
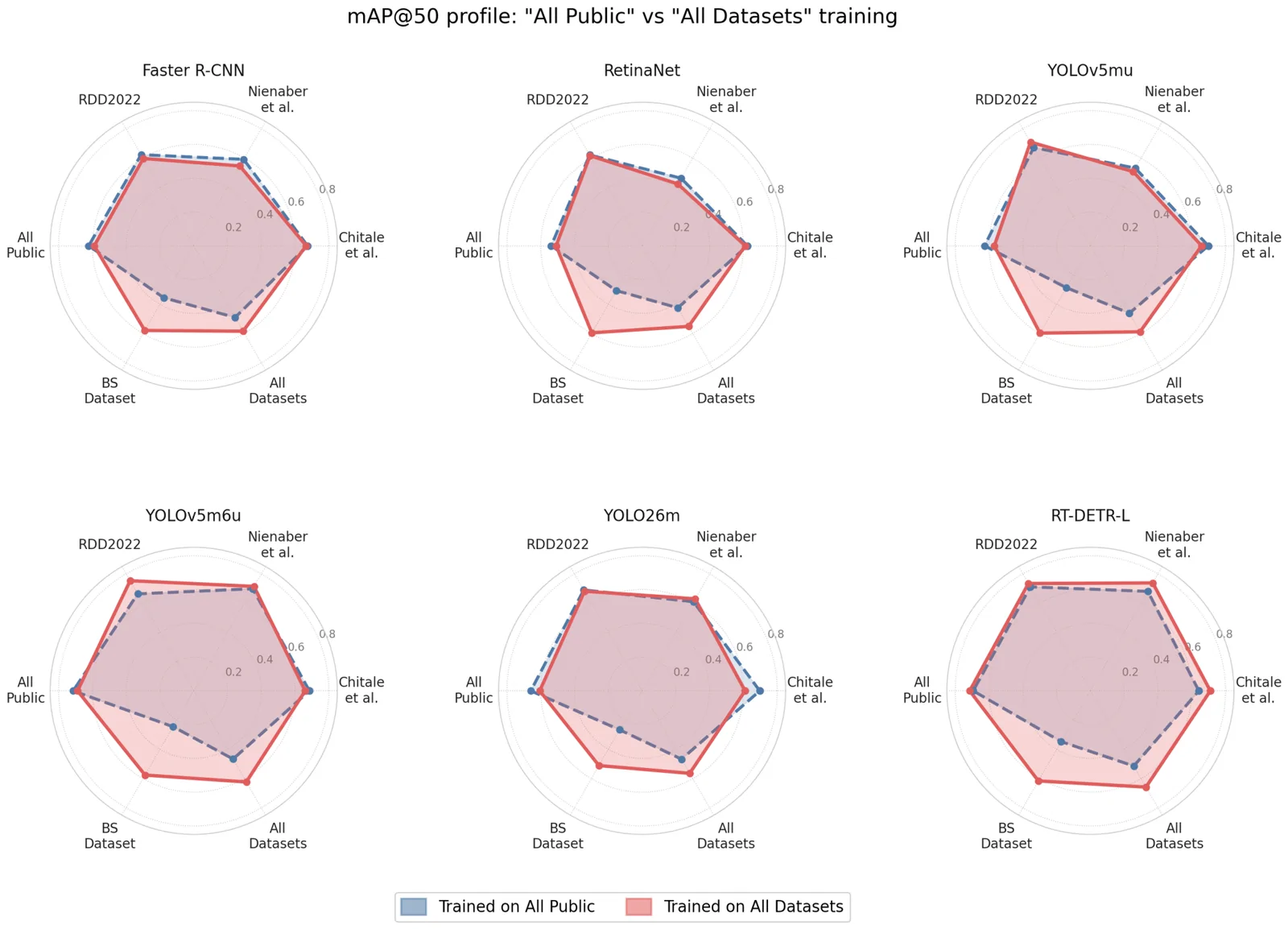
mAP@50: training result comparison between all public sets and all public + BS Dataset.

**Figure 12:**
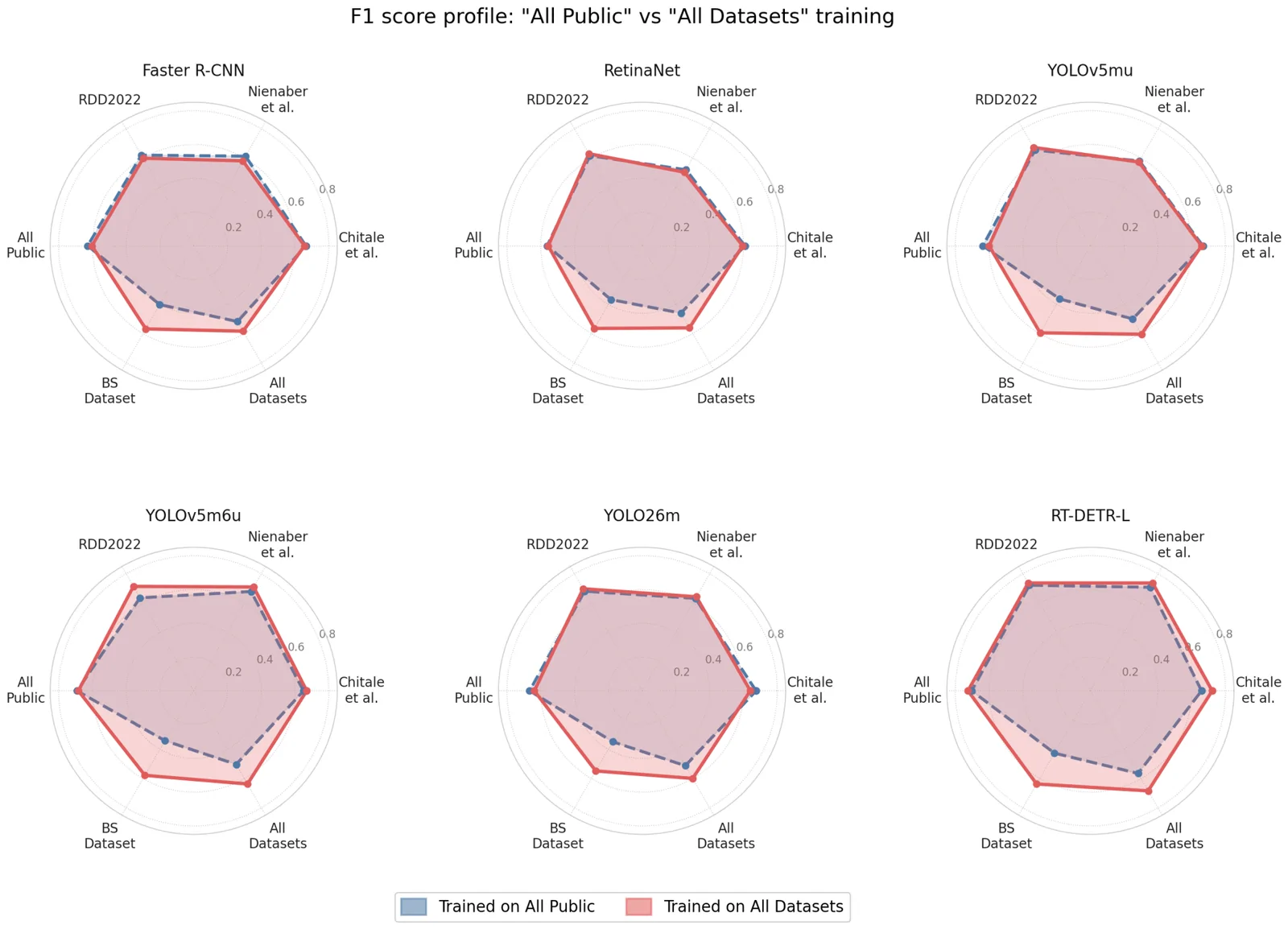
F1 score: training results comparison between all public sets and all public + BS Dataset.

Figure 8, Figure 9, Figure 10, Figure 11 and Figure 12 illustrates the prediction results of different models.

When comparing different model architectures, YOLO26m, despite its more recent anchor-free and NMS-free design, performs worse than the older YOLOv5mu model, particularly compared to the m6u variant that uses a higher input resolution of 1280 × 1280. We believe this is due to the simpler and more stable architecture of YOLOv5 combined with the benefit of higher input resolution, which is especially important for pothole detection, where objects are typically small compared to standard benchmarks such as MS COCO [27] and Pascal VOC [31].

Faster R-CNN and RetinaNet generally achieve lower results compared to newer one-stage and transformer-based approaches like YOLOv5m6u and RT-DETR-L, although they remain stable across all benchmarks, and overall tend to perform very similarly to the YOLO variants with smaller input sizes like YOLOv5mu and YOLO26m. We believe that the larger input resolution of 800 × 800 pixels provides an advantage for pothole detection, particularly for small objects. This is especially evident in the datasets of S. Nienaber et al. and in our BS Dataset, where many potholes occupy only a limited portion of the image.

## 6. Future Work

Several directions remain open for future research. This work has shown that building an efficient, general, and comprehensive dataset for road pothole detection is a challenging task, mainly due to variability in real-world conditions and the complexity of data collection.

First, the dataset can be further extended using the developed smartphone application by acquiring data from additional cities, as well as under different weather and seasonal conditions. This would improve the generalization capabilities of models trained on the dataset.

Second, the annotation schema could be expanded to include more types of road damage, not only potholes. This would allow the dataset to support a broader range of road distress detection tasks, potentially reaching a scope similar to datasets such as RDD2022.

Third, the sensing capabilities of the acquisition system create opportunities for multimodal approaches. Future work could explore combining visual data with vehicle telemetry information, such as inertial measurements, vehicle and wheel speeds, and GPS data, to develop more robust detection systems based on sensor fusion techniques.

Fourth, the data acquisition pipeline itself could be improved by integrating lightweight detection models directly into the application. This could reduce reliance on manually tuned thresholds for collision detection and make the data collection process more efficient and autonomous.

Overall, while the BS Dataset represents a significant contribution to pothole detection research, its combination of visual and sensor data offers further opportunities for advancing multimodal and real-world intelligent road monitoring systems.

## 7. Conclusions

This work presented a methodology for collecting and annotating multimodal data for road pothole detection through a custom smartphone application and validated its effectiveness through the creation and analysis of the BS Dataset. The proposed acquisition framework enables the synchronized collection of road images and vehicle telemetry data under real driving conditions, providing a basis for the development and evaluation of pothole detection systems.

Experimental results demonstrated the usefulness of the collected data for training object detection models.

In particular, the best-performing model, RT-DETR-L, trained using the proposed dataset, achieved a mAP@50 of 0.658 and an F1-score of 0.684 on the global test set.

The results demonstrated that models trained on the proposed BS Dataset consistently achieved higher detection performance on multiple publicly available pothole datasets, indicating strong cross-dataset generalization capabilities. In particular, RT-DETR-L achieved increases in mAP@50 of 0.068, 0.058, and 0.044 on the datasets of Chitale et al., S. Nienaber et al., and RDD2022, respectively.

Furthermore, the dataset contains 17,553 annotated images and 24,704 pothole instances, offering a substantial resource for future research.

The collected data were acquired in diverse environmental and operational conditions, including variations in illumination, pavement quality, traffic, camera orientation, and vehicle motion. These characteristics make the dataset representative of real-world scenarios and allow researchers to evaluate model performance under conditions that are closer to practical deployment than those typically found in controlled experimental settings. As a result, the proposed methodology contributes to narrowing the gap between laboratory validation and real-road applications.

In addition, the sensing capabilities of the PotholeByCamera App provide both visual information and telemetry measurements. This multimodal structure enables the development of approaches that combine image-based analysis with vehicle-dynamic responses, potentially improving detection accuracy, enhancing robustness against challenging environmental conditions, and supporting the assessment of pavement hazard severity in terms of driving safety, vehicle wear, and road maintenance requirements. Consequently, the dataset can facilitate future research on intelligent pavement monitoring and maintenance-support systems.

## Figures and Tables

**Table 1:** Publicly available datasets for road hazard detection including the road pothole category.

Dataset	Total Images	Pothole Images	Content	Key Limitations
RDD2022 [19]	47,420	1888	Large-scale, multinational coverage, detailed multi-class annotations, multiple acquisition methods (vehicles, drones, motorbikes)	High class imbalance, low coverage of pothole class
S. Nienaber et al. [8]	8454	2405	Vehicle-mounted driving perspective, accurate single-class annotations of road potholes, high-resolution images	Low variability, mainly open roads with low traffic
Chitale et al. [15]	1243	1243	Web scraping, high variability, single class annotation of road potholes, mixed sources	Inconsistent image size, low image quality, inconsistent domain

**Table 2:** Instances of the Android *Sensor* class used in the development of the smartphone application.

Sensor	Description	Unit
TYPE_ACCELEROMETER	Acceleration along the x-axis (including gravity)	m/s^2^
	Acceleration along the y-axis (including gravity)	m/s^2^
	Acceleration along the z-axis (including gravity).	m/s^2^
TYPE_LINEAR_ACCELERATION	Acceleration along the x-axis (excluding gravity)	m/s^2^
	Acceleration along the y-axis (excluding gravity)	m/s^2^
	Acceleration along the z-axis (excluding gravity)	m/s^2^
TYPE_ROTATION_VECTOR	Rotation vector component along the x axis (x∗sin(θ/2))	Unitless
	Rotation vector component along the y axis (y∗sin(θ/2))	Unitless
	Rotation vector component along the z axis (z∗sin(θ/2))	Unitless
	Scalar component of the rotation vector ((cos(θ/2))	Unitless

**Table 3:** Project relevant specifications of the Samsung Galaxy A55.

OS	Android 14
CPU	4× 2.75 GHz Cortex-A78 + 4× 2.0 GHz Cortex-A55
RAM	8 GB
ROM	256 GB
Main Camera	50 MP
Optical Stabilizer	Yes
Max video resolution	4K (2160p)
Video framerate	30 fps
Sensors	Accelerometer, Gyroscope, Magnetometer, GPS
Battery capacity	5000 mAh

**Table 4:** List of tested object detection models.

Name	Short Name	Input Size	No. Parameters
Faster R-CNN ResNet50 FPN v2	faster r-cnn	800 × 800 × 3	43.2M
RetinaNet ResNet50 FPN v2	retinanet	800 × 800 × 3	36M
YOLOv5mu	yolov5mu	640 × 640 × 3	21.2M
YOLOv5m6u	yolov5m6u	1280 × 1280 × 3	35.7M
YOLO26m	yolo26m	640 × 640 × 3	20.4M
RT-DETR (Large)	rt-detr-l	1024 × 1024 × 3	32.8M

**Table 5:** Dataset splitting. Each subset is randomly divided into training, validation and test sets with ratios 70%-20%-10%.

Dataset	Train Count	Valid Count	Test Count	Total
Chitale et al. [15]	870	248	125	1243
S. Nienaber et al. (simplex+complex) [8]	1682	481	241	2404
RDD2022 (pothole) [19]	1321	377	190	1888
BS Dataset (subsample + stratification)	4679	1469	653	6801
all public (Chitale et al. [15] + S. Nienaber et al. [8] + RDD2022 [19])	3873	1106	556	5535
all datasets (public + BS)	8552	2575	1209	12,336

**Table 6:** Testing results of object detection methods on different training datasets. Each model was trained and tested on different sets.

		Test Set																	
		Chitale et al.			S. Nienaber et al.			RDD2022			All Public			BS Dataset (Ours)			All Datasets		
Model	Train Dataset	mAP@50	mAP@50:95	F1	mAP@50	mAP@50:95	F1	mAP@50	mAP@50:95	F1	mAP@50	mAP@50:95	F1	mAP@50	mAP@50:95	F1	mAP@50	mAP@50:95	F1
**faster r-cnn**	**Chitale et al.**	0.589	0.239	0.599	0.134	0.054	0.245	0.141	0.055	0.261	0.275	0.11	0.357	0.172	0.067	0.285	0.222	0.087	0.32
	**S. Nienaber et al.**	0.029	0.011	0.074	0.561	0.233	0.589	0.017	0.004	0.071	0.267	0.11	0.363	0.06	0.014	0.167	0.166	0.061	0.265
	**RDD2022**	0.245	0.093	0.323	0.083	0.02	0.19	0.558	0.248	0.583	0.237	0.091	0.333	0.283	0.111	0.363	0.261	0.101	0.351
	**all public**	**0.674**	**0.287**	**0.664**	**0.591**	**0.234**	**0.613**	**0.623**	**0.287**	**0.62**	**0.622**	**0.258**	**0.628**	0.355	0.14	0.402	0.49	0.199	0.518
	**BS Dataset (ours)**	0.161	0.058	0.272	0.13	0.035	0.265	0.347	0.143	0.431	0.179	0.062	0.301	**0.601**	**0.277**	**0.578**	0.395	0.17	0.454
	**all datasets**	0.666	0.283	0.656	0.546	0.211	0.581	0.597	0.27	0.599	0.589	0.242	0.603	0.579	0.272	0.568	**0.584**	**0.255**	**0.584**
**retinanet**	**Chitale et al.**	0.596	0.25	0.587	0.206	0.086	0.31	0.204	0.072	0.296	0.326	0.131	0.399	0.193	0.067	0.272	0.258	0.099	0.333
	**S. Nienaber et al.**	0.026	0.01	0.06	0.433	0.169	0.496	0.029	0.007	0.099	0.205	0.077	0.305	0.039	0.007	0.115	0.111	0.036	0.205
	**RDD2022**	0.263	0.102	0.302	0.08	0.024	0.173	0.557	0.238	0.559	0.241	0.094	0.31	0.275	0.108	0.344	0.258	0.101	0.328
	**all public**	**0.623**	**0.267**	**0.609**	**0.462**	**0.179**	**0.52**	**0.621**	0.258	0.616	**0.538**	**0.217**	**0.562**	0.306	0.11	0.368	0.424	0.164	0.46
	**BS Dataset (ours)**	0.256	0.085	0.318	0.109	0.025	0.195	0.402	0.158	0.463	0.211	0.067	0.302	0.557	0.24	0.54	0.385	0.153	0.436
	**all datasets**	0.611	0.258	0.596	0.423	0.151	0.503	0.616	**0.267**	**0.63**	0.509	0.199	0.556	**0.595**	**0.262**	**0.565**	**0.551**	**0.229**	**0.561**
**yolov5mu**	**Chitale et al.**	0.642	0.286	0.617	0.187	0.06	0.291	0.175	0.065	0.266	0.327	0.13	0.402	0.209	0.087	0.298	0.272	0.11	0.354
	**S. Nienaber et al.**	0.021	0.007	0.053	0.442	0.161	0.506	0.001	0	0.016	0.231	0.094	0.327	0.075	0.021	0.144	0.147	0.05	0.232
	**RDD2022**	0.052	0.017	0.123	0.006	0.001	0.029	0.421	0.168	0.432	0.107	0.041	0.185	0.137	0.042	0.224	0.113	0.038	0.199
	**all public**	**0.698**	**0.322**	**0.666**	**0.531**	**0.215**	**0.58**	0.672	0.318	0.655	**0.627**	**0.281**	**0.637**	0.286	0.115	0.363	0.461	0.198	0.5
	**BS Dataset (ours)**	0.074	0.021	0.171	0.082	0.021	0.178	0.21	0.079	0.32	0.103	0.032	0.209	0.543	0.261	0.564	0.327	0.146	0.405
	**all datasets**	0.658	0.295	0.658	0.507	**0.215**	0.573	**0.709**	**0.337**	**0.673**	0.569	0.252	0.6	**0.597**	**0.285**	**0.595**	**0.588**	**0.27**	**0.605**
**yolov5m6u**	**Chitale et al.**	0.644	0.302	0.634	0.303	0.117	0.386	0.139	0.056	0.229	0.388	0.168	0.44	0.161	0.056	0.239	0.264	0.106	0.339
	**S. Nienaber et al.**	0.02	0.007	0.054	0.59	0.253	0.593	0.014	0.002	0.061	0.316	0.152	0.402	0.092	0.027	0.192	0.185	0.071	0.275
	**RDD2022**	0.102	0.034	0.212	0.056	0.012	0.126	0.582	0.276	0.577	0.201	0.08	0.288	0.169	0.064	0.252	0.176	0.068	0.265
	**all public**	**0.685**	**0.314**	0.65	0.698	0.328	0.679	0.663	0.315	0.636	**0.714**	**0.353**	**0.691**	0.245	0.1	0.341	0.466	0.208	0.503
	**BS Dataset (ours)**	0.08	0.02	0.171	0.108	0.03	0.206	0.153	0.06	0.255	0.117	0.035	0.214	0.563	0.277	0.564	0.336	0.153	0.418
	**all datasets**	0.66	0.313	**0.669**	**0.714**	**0.35**	**0.71**	**0.753**	**0.363**	**0.714**	0.688	0.33	0.683	**0.576**	**0.287**	**0.578**	**0.623**	**0.303**	**0.637**
**yolo26m**	**Chitale et al.**	0.57	0.238	0.571	0.152	0.052	0.238	0.16	0.058	0.277	0.284	0.112	0.358	0.157	0.065	0.243	0.217	0.086	0.293
	**S. Nienaber et al.**	0.029	0.012	0.064	0.584	0.248	0.588	0.01	0.001	0.041	0.292	0.128	0.384	0.086	0.023	0.166	0.185	0.07	0.27
	**RDD2022**	0.158	0.054	0.242	0.018	0.003	0.05	0.613	0.274	0.627	0.197	0.078	0.284	0.171	0.056	0.246	0.181	0.066	0.261
	**all public**	**0.697**	**0.326**	**0.675**	0.609	0.259	0.632	**0.69**	**0.328**	0.681	**0.659**	**0.312**	**0.666**	0.266	0.094	0.348	0.467	0.196	0.512
	**BS Dataset (ours)**	0.085	0.023	0.2	0.129	0.036	0.235	0.154	0.06	0.245	0.121	0.036	0.224	0.503	0.235	0.54	0.307	0.13	0.394
	**all datasets**	0.61	0.275	0.641	**0.629**	**0.286**	**0.643**	0.681	0.325	**0.698**	0.606	0.274	0.638	**0.511**	**0.238**	**0.548**	**0.564**	**0.258**	**0.599**
**rt-detr-l**	**Chitale et al.**	0.623	0.299	0.634	0.294	0.121	0.411	0.186	0.046	0.303	0.377	0.157	0.457	0.18	0.06	0.283	0.273	0.105	0.373
	**S. Nienaber et al.**	0.042	0.018	0.083	0.695	0.312	0.699	0.068	0.018	0.164	0.382	0.184	0.474	0.105	0.029	0.204	0.228	0.089	0.329
	**RDD2022**	0.308	0.11	0.362	0.109	0.031	0.192	0.678	0.331	0.698	0.309	0.122	0.394	0.286	0.107	0.374	0.288	0.11	0.376
	**all public**	0.642	0.288	0.658	0.68	0.297	0.708	0.711	0.33	0.722	0.694	0.327	0.703	0.347	0.131	0.426	0.514	0.216	0.565
	**BS Dataset (ours)**	0.175	0.051	0.269	0.097	0.024	0.195	0.295	0.12	0.389	0.169	0.052	0.273	0.59	0.285	0.604	0.376	0.166	0.458
	**all datasets**	**0.71**	**0.349**	**0.721**	**0.738**	**0.361**	**0.737**	**0.734**	**0.357**	**0.737**	**0.715**	**0.349**	**0.725**	**0.616**	**0.313**	**0.637**	**0.658**	**0.325**	**0.684**

## Data Availability

The data presented in this study are available on request from the corresponding author. The dataset is property of Bridgestone Europe NV/SA—Italian Branch Technical Center Europe. The dataset contains sensitive information about public road images, vehicle license plates, private institutions and facilities, and identifiable human faces. Due to privacy, confidentiality, and data protection obligations, including compliance with applicable GDPR regulations, the dataset is not publicly available. Access may be granted only for research purposes and subject to approval by the data owner and the execution of appropriate data protection and non-disclosure agreements.

## References

[1] Hägg J. (2025). Potholes: Sweden, Europe, USA. https://www.niradynamics.com/latest/potholes-sweden-europe-usa.

[2] Fan R., Guo S., Wang L., Bocus M. (2023). Computer-Aided Road Inspection: Systems and Algorithms.

[3] Wu C., Wang Z., Hu S., Lepine J., Na X., Ainalis D., Stettler M. (2020). An Automated Machine-Learning Approach for Road Pothole Detection Using Smartphone Sensor Data. Sensors.

[4] Kulkarni A., Mhalgi N., Gurnani S., Giri N. (2014). Pothole Detection System using Machine Learning on Android. Int. J. Emerg. Technol. Adv. Eng..

[5] Bansal K., Mittal K., Ahuja G., Singh A., Gill S.S. (2020). DeepBus: Machine learning based real time pothole detection system for smart transportation using IoT. Internet Technol. Lett..

[6] Alleva L., Bortolotto V., Benedetti R., Pascucci M. (2024). Method and System for Detecting and Locating Obstacles/Elements on Road Pavement that Are Dangerous or Potentially Dangerous to Tire and/or Vehicle Integrity.

[7] Alleva L., Boldrini A., Massimilla M., Nicolosi V., Iannantuono A. (2024). Method and Related System for Estimating the International Roughness Index of a Road Segment.

[8] Nienaber S., Booysen M.T., Kroon R. Detecting Potholes Using Simple Image Processing Techniques and Real-World Footage. Proceedings of the South African Transport Conference.

[9] Tedeschi A., Benedetto F. (2017). A real-time automatic pavement crack and pothole recognition system for mobile Android-based devices. Adv. Eng. Inform..

[10] Huang D., Shan C., Ardabilian M., Chen L. (2011). Local Binary Patterns and Its Application to Facial Image Analysis: A Survey. IEEE Trans. Syst. Man Cybern. Part C.

[11] Hoang N.D. (2018). An Artificial Intelligence Method for Asphalt Pavement Pothole Detection Using Least Squares Support Vector Machine and Neural Network with Steerable Filter-Based Feature Extraction. Adv. Civ. Eng..

[12] Boser B., Guyon I., Vapnik V. A Training Algorithm for Optimal Margin Classifier. Proceedings of the COLT92: 5th Annual Workshop on Computational Learning Theory.

[13] Samanta R., Sadasivan A., Kavitha M.S., Balasubramanian S. (2026). Artificial Intelligence for Road Anomaly Detection: A Review. WIREs Data Min. Knowl. Discov..

[14] Zhao Z.Q., Zheng P., Xu S.T., Wu X. (2019). Object Detection With Deep Learning: A Review. IEEE Trans. Neural Netw. Learn. Syst..

[15] Chitale P.A., Kekre K.Y., Shenai H.R., Karani R., Gala J.P. Pothole Detection and Dimension Estimation System using Deep Learning (YOLO) and Image Processing. Proceedings of the 2020 35th International Conference on Image and Vision Computing New Zealand (IVCNZ).

[16] Maeda H., Sekimoto Y., Seto T., Kashiyama T., Omata H. (2018). Road damage detection and classification using deep neural networks with smartphone images. Comput.-Aided Civ. Infrastruct. Eng..

[17] Angulo A., Vega Fernandez J., Aguilar Lobo L., Natraj S., Ochoa-Ruiz G. (2019). Road Damage Detection Acquisition System Based on Deep Neural Networks for Physical Asset Management.

[18] Pereira V., Tamura S., Hayamizu S., Fukai H. A Deep Learning-Based Approach for Road Pothole Detection in Timor Leste. Proceedings of the 2018 IEEE International Conference on Service Operations and Logistics, and Informatics (SOLI).

[19] Arya D., Maeda H., Ghosh S.K., Toshniwal D., Sekimoto Y. (2024). RDD2022: A multi-national image dataset for automatic road damage detection. Geosci. Data J..

[20] Girshick R., Donahue J., Darrell T., Malik J. Rich feature hierarchies for accurate object detection and semantic segmentation. Proceedings of the 2014 IEEE Conference on Computer Vision and Pattern Recognition.

[21] Ren S., He K., Girshick R., Sun J., Cortes C., Lawrence N., Lee D., Sugiyama M., Garnett R. (2015). Faster R-CNN: Towards Real-Time Object Detection with Region Proposal Networks. Proceedings of the Advances in Neural Information Processing Systems.

[22] Liu W., Anguelov D., Erhan D., Szegedy C., Reed S., Fu C.Y., Berg A.C., Leibe B., Matas J., Sebe N., Welling M. (2016). SSD: Single Shot MultiBox Detector. Proceedings of the Computer Vision—ECCV 2016.

[23] Lin T.Y., Goyal P., Girshick R., He K., Dollár P. (2020). Focal loss for dense object detection. IEEE Trans. Pattern Anal. Mach. Intell..

[24] Redmon J., Divvala S., Girshick R., Farhadi A. You only look once: Unified, real-time object detection. Proceedings of the 2016 IEEE Conference on Computer Vision and Pattern Recognition (CVPR).

[25] Carion N., Massa F., Synnaeve G., Usunier N., Kirillov A., Zagoruyko S., Vedaldi A., Bischof H., Brox T., Frahm J.M. (2020). End-to-end object detection with transformers. Computer Vision – ECCV 2020.

[26] Arya D., Maeda H., Ghosh S.K., Toshniwal D., Omata H., Kashiyama T., Sekimoto Y. Crowdsensing-based road damage detection challenge (CRDDC’2022). Proceedings of the 2022 IEEE International Conference on Big Data (Big Data).

[27] Lin T.Y., Maire M., Belongie S., Hays J., Perona P., Ramanan D., Dollár P., Zitnick C.L., Fleet D., Pajdla T., Schiele B., Tuytelaars T. (2014). Microsoft COCO: Common objects in context. Computer Vision–ECCV 2014.

[28] Jocher G. (2020). Ultralytics YOLOv5. https://zenodo.org/records/7347926.

[29] Jocher G., Qiu J. (2026). Ultralytics YOLO26. https://github.com/ultralytics/ultralytics.

[30] Zhao Y., Lv W., Xu S., Wei J., Wang G., Dang Q., Liu Y., Chen J. DETRs Beat YOLOs on Real-time Object Detection. Proceedings of the 2024 IEEE/CVF Conference on Computer Vision and Pattern Recognition (CVPR).

[31] Everingham M., Van Gool L., Williams C.K.I., Winn J., Zisserman A. (2010). The Pascal Visual Object Classes (VOC) Challenge. Int. J. Comput. Vis..

